# eABR THR Estimation Using High-Rate Multi-Pulse Stimulation in Cochlear Implant Users

**DOI:** 10.3389/fnins.2021.705189

**Published:** 2021-07-29

**Authors:** Ali Saeedi, Ludwig Englert, Werner Hemmert

**Affiliations:** ^1^Department of Electrical and Computer Engineering, Technical University of Munich, Munich, Germany; ^2^Munich School of Bioengineering, Technical University of Munich, Garching, Germany; ^3^Munich School of Robotics and Machine Intelligence, Technical University of Munich, Munich, Germany

**Keywords:** multi-pulse stimulation, temporal integration, brainstem response, cochlear implants, threshold estimation, objective measure

## Abstract

We estimated the electrically-evoked auditory brainstem response thresholds (eABR THRs) in response to multi-pulses with high burst rate of 10,000 pulses-per-second (pps). Growth functions of wave eV amplitudes, root mean square (RMS) values, peak of phase-locking value (PLV), and the lowest valid data point (LVDP) were calculated in 1-, 2-, 4-, 8-, and 16-pulses conditions. The growth functions were then fitted and extrapolated with linear and exponential functions to find eABR THRs. The estimated THRs were compared to psychophysical THRs determined for multi-pulse conditions as well as to the clinical THRs measured behaviorally at the rate of 1,000 pps. The growth functions of features showed shallower growth slopes when the number of pulses increased. eABR THRs estimated in 4-, 8-, and 16-pulses conditions were closer to the clinical THRs, when compared to 1- and 2-pulses conditions. However, the smallest difference between estimated eABR THRs and clinical THRs was not always achieved from the same number of pulses. The smallest absolute difference of 30.3 μA was found for the linear fittings on growth functions of eABR RMS values in 4-pulses condition. Pearson’s correlation coefficients (PCCs) between eABR THRs and psychophysical THRs were significant and relatively large in all but 16-pulses conditions. The PCCs between eABR THRs and clinical THRs, however, were smaller and in less cases significant. Results of this study showed that eABRs to multi-pulse stimulation could, to some extent, represent clinical stimulation paradigms, and thus in comparison to single pulses, could estimate clinical THRs with smaller errors.

## Introduction

Appropriate adjustment of hearing threshold (THR) levels is important in cochlear implant (CI) fitting in order to make the best use of the limited dynamic range (DR) available in electric hearing. Precise THR estimation, especially for the first fit after implantation, can provide the user with improved perception of soft sounds, which in turn helps for a better speech perception (Holden et al., 2011; Rader et al., 2018). Setting the THRs too low or too high results in suboptimal usage of the available DR. In the former case, quiet sounds cannot be perceived and in the latter, the available DR is reduced and CI users might even perceive an irritating background noise (Busby and Arora, 2016). Clinically, THR levels are determined by direct adjustment/feedback from the implantee. The procedure of THR determination becomes hard if the implantee is unable to cooperate, e.g., infants or users with lack of proper communication. In such situations, objective estimation of THRs, where electrically-evoked objective measures of the auditory pathway are used, could be an alternative. Among these measures are electrically-evoked compound action potentials (eCAP, peripheral measure), electrically-evoked auditory brainstem responses (eABR), electrically-evoked auditory steady state response (eASSR), and cortically evoked potentials (CAEP) (central measures). The extent to which the THRs estimated in each of these measures correlate with the behavioral THRs are reported to be different, with generally better performance in central measures compared to peripheral ones [e.g., CAEP vs. eCAP in Abbas and Brown (2015)]. Although responses from higher auditory brain areas capture the THRs better, they are less suitable for THR estimates in newborns and young infants for two reasons: higher level potentials require attention (Picton et al., 1971; Picton and Hillyard, 1974), and the auditory pathway is not yet developed. Therefore, a compromise between more peripheral (e.g., eCAP) and more central auditory responses (e.g., CAEP) has to be found.

Electrically-evoked compound action potentials overestimate the behavioral THRs with moderate correlation when the behavioral stimulation rate is low, e.g., 250 pulses-per-second (pps; Miller et al., 2008) and correlation decreases when stimulation rates increase. This is due to the fact that in eCAP measurements single stimulation pulses of low stimulation rates are used, which do not reflect temporal integration effects observed when high rate pulse trains are used for clinical fittings. This limits the prediction power of the behavioral THRs (Miller et al., 2008; McKay et al., 2013; Mao et al., 2019). Conventional eABRs showed relatively high correlation with behavioral THRs when the behavioral stimulation rate is less than 500 pps, e.g., *r* = 0.89 at 10 pps (Hodges et al., 1994), *r* = 0.83 at 35–80 pps (Brown et al., 2000), *r* = 0.98 at 300 pps (Truy et al., 1998), and *r* = 0.84 and 0.74 at single pulses and pulse trains of 400 pps, respectively (Brown et al., 1999). However, the correlation between eABR THRs and behavioral THRs reported to decrease when the stimulation rate increased, e.g., *r* = 0.69 at 1,000 pps in Brown et al. (1999).

In central electrophysiological recordings (e.g., eASSR and CAEP), larger correlations were found compared to those reported in eCAP and eABR measurements. In an eASSR study, Hofmann and Wouters (2012) showed high correlations between eASSR THRs and behavioral THRs either for 40 pps pulse trains (*r* = 0.96) or 900 pps amplitude modulated (AM) and phase-width modulated (PWM) pulse trains (*r* = 0.96 and *r* = 0.96, respectively). In a CI study, Visram et al. (2015) recorded CAEPs in response to 50 ms pulse trains presented at 900 pps, and found high correlations between behavioral THRs and cortical THRs (*r* = 0.93). Using a phase-locking feature value for CAEP growth functions, Mao et al. (2019) showed high correlations between CAEP THRs and behavioral THRs (*r* = 0.979 in the standard Cz-to-mastoid montage and *r* = 0.96 in recordings from channels near the CI). Although cortical potentials (eASSR and CAEP) showed promising objective THR estimates, they have still limitations that restrict their applicability for clinical purposes. For instance, subjects should remain awake and as calm as possible during the cortical measurements, which restricts the method for infant CI users. Therefore, it remains worthwhile to introduce modifications to other established measures (e.g., eABR) with the aim of improving their functionality to achieve more accurate objective THR estimates.

Neurons would respond differently to stimuli with different parameters, such as pulse shape and stimulus frequency (Mahmud and Vassanelli, 2016). One modification to the conventional (single-pulse) eABR measurements could be employing multiple-pulse (MP) stimuli with the aim to account also for loudness integration, which is prominent for typical environmental- and speech sounds. Multi-pulse integration (MPI) suggests that at a fixed stimulation rate, the detection THRs improves when the number of pulses (or equivalently the stimulation duration) increases. Compared to stimulation rates below 1,000 pps, the MPI slopes for rates above 1,000 pps are steeper in guinea pigs (Kang et al., 2010; Zhou et al., 2015) as well as in humans (Shannon, 1985; McKay and McDermott, 1998; Zhou et al., 2012, 2015; Carlyon et al., 2015). Carlyon et al. (2015) found that when the number of pulses increased from 1 to 16, MPI slopes decreased by about 0.68 and 1.33 dB/doubling the number of pulses, for rates of 500 and 3,500 pps, respectively. These drops are equivalent to. Obando Leitón (2019) found that at rates of 1,500 and 18,000 pps, MPI slopes dropped 3.44 and 5.43 dB per tenfold increase of the number of pulses, which correspond to drops of 1.03 and 1.63 dB per doubling the number of pulses, respectively. In a previous study (Saeedi and Hemmert, 2020), we measured behavioral THRs and MCLs as well as eABRs in response to 1-, 2-, 4-, 8-, and 16-pulses stimuli at the rate of 10,000 pps. MPs were constructed by assembling single-pulses closely together to make the stimuli more representative of high-rate clinical stimulation paradigms. We found behavioral MPI slopes of −1.30 and −0.93 dB/doubling of the number of pulses for behavioral THRs and MCLs, respectively.

Our previous study (Saeedi and Hemmert, 2020) aimed to assess temporal effects and efficiency of MPs in eABR. We found that eABR morphology in response to MP stimuli did not differ from those to conventional single-pulse stimuli. It was also shown that introducing more pulses led to larger wave eV amplitudes up to a certain subject-specific number of pulses. The saturation of the growth function was attributed to the destructive interference of the eABRs to later pulses in a pulse train, where time-shifted peaks and troughs of later pulses suppressed those of earlier pulses. This study aimed to (1) investigate how features extracted from the eABRs in response to MPs grow and (2) see how well the estimated THRs in MP conditions correlate with the behavioral THRs. We measured psychophysical THRs at MP conditions as well as clinical THRs. We also measured eABRs to MP stimulations from 5 to 95% of the corresponding DRs. Then, we calculated growth functions of eABR wave eV amplitudes, root mean square (RMS) values, peak phase-locking value (peak PLV), and the lowest valid data point (LVDP). We fitted and extrapolated the growth functions of these features with a linear and an exponential fitting function (FF) to estimate eABR THRs. The estimated eABR THRs were then compared to those from psychophysical measurements as well as to the clinical THRs. We assumed that eABR THRs in response to MPs could estimate clinical THRs more accurately, as in our previous study (Saeedi and Hemmert, 2020) psychophysical THRs tended to approach the clinical THRs when the number of pulses increased from 1 to 16.

## Materials and Methods

A total of thirteen ears from nine CI users (three males, mean age: 50.6 years) implanted with MED-EL CIs were measured. Demographic information of the participants is available in Table 1. Participants signed an informed consent and received a compensation fee for their participation. The study was approved by the Medical Ethics Committee of the Klinikum rechts der Isar, Munich.

**TABLE 1:** Demographic information of cochlear implant (CI) participants.

Subject	Side(s)	Age range (years)	Etiology	Deafness dur. (years)	CI experience (years)	CI type	Electrode
S1	L	50–55	Inherited OM	49	4	Co	6
S2	L, R	56–60	Congenital	56	12, 10	P, So	6, 4
S3	L, R	60–65	Unknown	22	4.5, 5	So, So	4, 6
S4	L, R	56–60	Unknown	56	11, 10	P, P	6, 7
S8	L	40–45	Congenital	42	5	Co	4
S10	L, R	75–80	Unknown	30, 20	20, 12	Sy, P	4, 5
S12	R	20–25	Meningitis	22	10	So	5
S13	L	40–45	OM	40	3	Sy	7
S14	R	35–40	Inherited OM	31	6	Co	4

*OM, otitis Media; Co, concerto; P, pulsar; So, sonata; Sy, synchrony.*

### Stimuli

**FIGURE 1:**
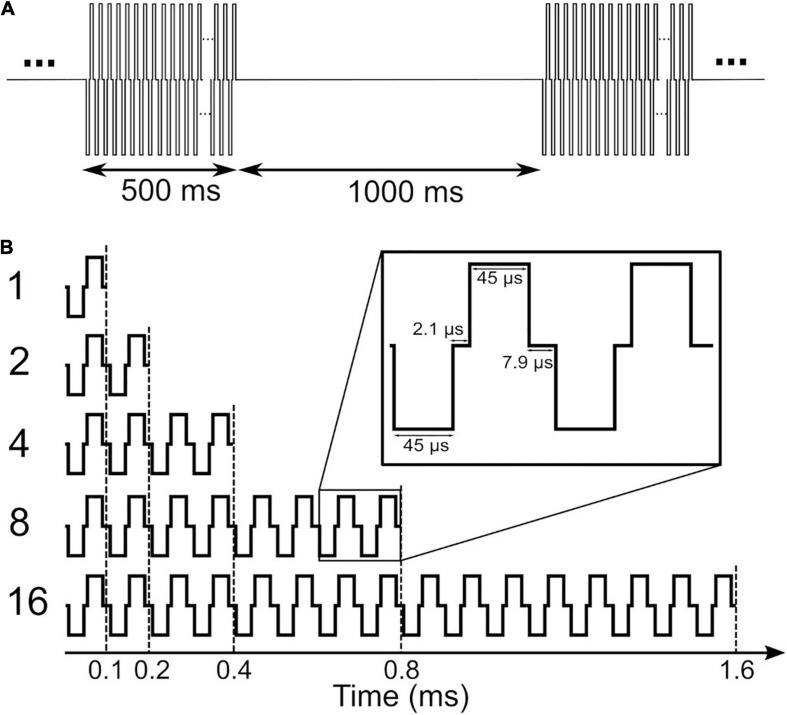
Stimuli used in clinical measurements **(A)** and in electrically-evoked auditory brainstem response (eABR) measurements **(B)**. Stimuli in panel **(B)** were presented at a repetition rate of 37 pps.

A schematic of the stimuli used in this study is depicted in Figure 1. Stimuli in clinical measurements consisted of 500 ms pulse trains with a stimulation rate of 1,000 pps followed by a 1,000 ms pause (Figure 1A). In clinical measurements, single pulses were anodic-first charge-neutral biphasic pulses with 45 μs phase width and 2.1 μs inter-phase gap. Stimuli for eABR measurements were same as in our previous work (Saeedi and Hemmert, 2020), where electrical multi-pulse (MP) trains of 1-, 2-, 4-, 8-, and 16-pulses were employed (Figure 1B). Multi-pulses were assembled by concatenating single pulses. Properties of single pulses in the eABR measurements were identical to those in clinical measurements. Additionally, an inter-pulse gap of 7.9 μs was used to achieve a pulse period of 100 μs, which corresponds to a burst rate of 10,000 pps. Stimuli for eABR measurements were delivered to an electrode in the middle of the array.

### Pretest

Psychophysical thresholds (THRs) and the most comfortable levels (MCLs) for eABR and clinical measurements were adjusted by the subjects in four different sessions during the same day. In psychophysical measurements, all MP conditions of THR/MCL were presented separately; e.g., all MP THRs were measured in one session and all MP MCLs were measured in another session. Each THR/MCL was measured three times; one trial round and two main rounds. Only results of the main rounds were used for further analysis. The same stimuli employed in psychophysical measurements were later used in eABR measurements. The clinical THRs measured in pretest sessions were compared to the estimated eABR THRs. More details on psychophysical measurements can be found in Saeedi and Hemmert (2020).

### eABR Stimulation

In order to estimate eABR THRs in each MP condition, the corresponding eABR amplitude growth functions (AGFs) were measured. Stimuli with amplitudes of 5–95% of the DR with steps of 10% were used. For two subjects, high stimulation amplitudes stimulated the facial nerve and thus resulted in artifact-corrupted eABRs. These conditions were excluded from further analysis. In most of the subjects, no clean eABR was observed at low stimulation amplitudes, e.g., 5% of the DR. When less than four points remained in the AGFs, extra stimuli were used to add more points to the AGFs and thus to make the eABR estimation procedure feasible. Stimulation scripts were developed and compiled in MATLAB 9.6.0.1072779 (2019a) installed on a personal computer. The compiled scripts were then delivered through a National Instrument (NI) I/O card to a research interface box (RIB II), manufactured by the University of Innsbruck, Innsbruck, Austria. The RIB II conveyed the stimulation pulse sequences to the internal part of the implant via an induction coil.

### eABR Recording

Electrically-evoked auditory brainstem responses were measured differentially from surface electrodes glued on the skin. Raw eABRs were recorded with a Biopac®, MP36 system (California, United States) with a sampling rate of 100 kHz, 24-bit A/D converter and amplifier gain set to 1,000. eABRs in MP conditions were measured in separate sessions. Measurements in each MP condition were randomized through the stimulation amplitudes (5–95% of the DR, maximum of 10 conditions). For each stimulation amplitude of MP conditions, 2,184 epochs were recorded, each of which had a duration of 27 ms (totally about 59 s). Subjects were sitting or laying on a comfortable couch during the eABR recordings. They were asked to close their eyes, not to blink, and stay as calm as possible during stimulation to minimize myogenic/muscle artifacts. Subjects were allowed to move freely between two consecutive measurements. Regular breaks were made and subjects were also free to request a break or to terminate the experiment at any time during the measurement. In order to achieve a low recording electrode impedance, the skin beneath electrodes was cleaned with alcohol swabs, and scrubbed by subjects themselves as thorough as they possibly could. Conductive gel was used to minimize the impedance between the electrodes and the skin. Electrode impedances were monitored by the recording setup and were below 10 kΩ during the whole measurement time.

### eABR THR Estimation

Raw eABRs were processed offline using MATLAB. The procedure of eABR processing included stimulus onset detection, electrical artifact suppression by exponential fitting, band-pass filtering, and weighted averaging. We used weighted non-stationary fixed multi-points (WNSFMP) averaging method, introduced by Silva (2009), to minimize the noise mainly originated in myogenic activities as well as spontaneous activity of the brain (e.g., EEG). The WNSFMP method is a powerful method to estimate the noise even in non-stationary situations such as auditory processing. The eABR processing steps were described in detail in Saeedi and Hemmert (2020). The WNSFMP method provides post-average residual noise (RN) estimation. In this study, eABR amplitude variances were estimated as ${\hat{\sigma }}_{\text{amp}}^{2}=2{\hat{\sigma }}_{\text{RN}}^{2}$, as in Undurraga et al. (2013). Only eABR waves eV with amplitudes greater than $\sqrt{2}{\hat{\sigma }}_{\text{RN}}$ were accepted as valid responses. One can think of increasing the number of averages to improve the signal-to-noise ratio. However, significantly larger numbers of averages beyond 2,000 are not practicable due to the long measurement times. Therefore, eABRs with low amplitudes are stronger affected by noise. This is also true for longer stimulation durations of MPs, which would consider temporal integration effects better. Long stimuli smear out the eABR responses and reduce their amplitudes due to destructive interferences, as described in Saeedi and Hemmert (2020).

Four features were used for eABR THR estimation: wave eV amplitudes, RMS values, peaks of PLV (Mao et al., 2018, 2019), and the LVDP, where still a valid wave eABR eV could be detected (LVDP). All four features were calculated on the block average of clean epochs. eABR wave eV amplitude was defined as the difference between peak eV and the following trough amplitude. eABR RMS value was calculated for valid eABR responses in a time window from 2.5 to 6.5 ms after stimulus onset. eABR peak PLV was calculated by first taking the short-time Fourier transform (STFT) on a post-stimulus window from 2.5 to 6.5 ms after stimulus onset. A hamming window of length 150 samples and an overlap of 100 samples were used for the calculation of the STFT. The phase-locking spectrograms were calculated at 270 frequencies linearly spaced between 300 and 3,000 Hz, by calculating the phase of each time-frequency point of the STFT (θ_*i*_(*t*,*f*)) and then applying the formula in Eq. 1 to calculate the phase-locking spectrogram (Mardia, 2014). The peak PLV was the maximum value in the PLV spectrogram.

(1) $$\mathrm{PLV}\left(t,f\right)=\frac{1}{N}\sqrt{{\left[\sum_{i=1}^{N}\cos\left(\theta_{i}\left(t,f\right)\right)\right]}^{2}+{\left[\sum_{i=1}^{N}\sin\left(\theta_{i}\left(t,f\right)\right)\right]}^{2}}$$

(2) $$f\left(x\right)=a\left(1-e^{-\frac{x-b}{c}}\right)$$

(3) f(x)=a(x-b)

**FIGURE 2:**
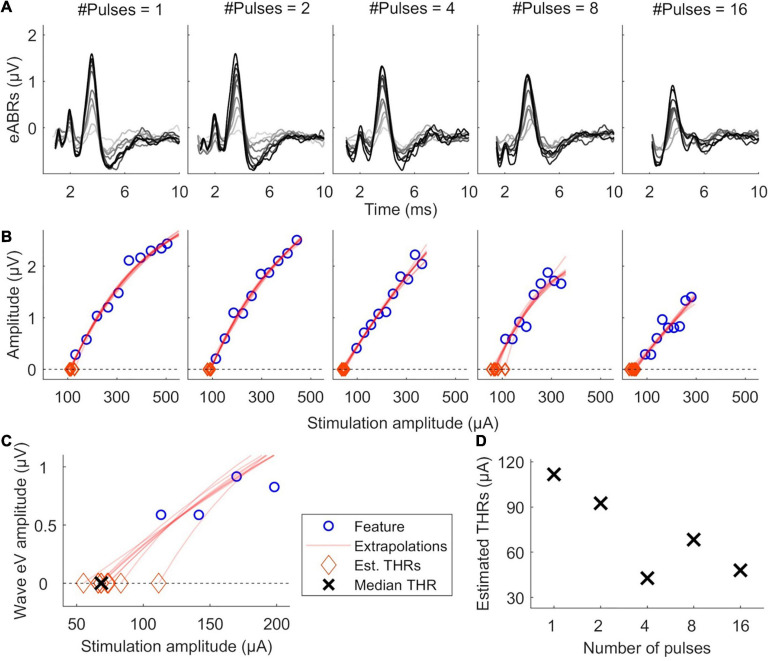
Illustration of eABR threshold (THR) estimation for subject S8L. **(A)** valid eABRs in each MP condition. In each panel, eABRs to different stimulation amplitudes are plotted. **(B)** corresponding growth functions (blue circles) in panel **(A)**, growth function fittings (red lines) and eABR THR estimations (red diamonds). **(C)** Details of eABR estimation from B (8-pulses condition). The “ × ” shows the median of estimated eABR THRs (diamonds). **(D)** Estimated eABR THRs as a function of number of pulses.

Figure 2 illustrates the estimation of eABR THR. For each MP condition, valid points of the features’ growth functions were fitted with a linear or an exponential growth function. THRs were estimated using the median from subsamples of growth functions, where one data point was excluded from the fit and the THR was extrapolated from the remaining data points. THRs were extrapolated with an exponential and a linear function, as described in Eqs. 2 and 3, respectively. In Eq. 2, *f*(*x*) represents a feature, *x* represents the stimulation amplitude in %DR, *a* the asymptote, *b* the *x*-intercept and *c* the exponential growth. In Eq. 3, *a* represents the growth slope and *b* the *x*-intercept. Fitted functions were extrapolated to intersect the *x*-axis, where the features are zero. The intersection point was assumed as the eABR THR. Two criteria were considered in THR estimation: (1) the 25th percentile of the THRs from the leave-one-out method is positive; (2) the median (50th percentile) is bigger than the difference between 75th and 25th percentiles. The first criterion helped to remove negative THR estimates and the second criterion provided an unbiased estimation.

### Statistical Analysis

Repeated-measures analysis of variance (ANOVA) was used to statistically test the effect of the number of pulses. Fisher’s *r* to z transformation and z-test statistics were employed to compare of Pearson correlation coefficients (PCCs). MATLAB 9.6.0.1072779 (2019a) was used for all statistical analysis. For pairwise comparisons, Bonferroni corrected post hoc analysis was used. The significance level was set to α = 0.05 for all analysis.

## Results

### Psychophysical Thresholds

**FIGURE 3:**
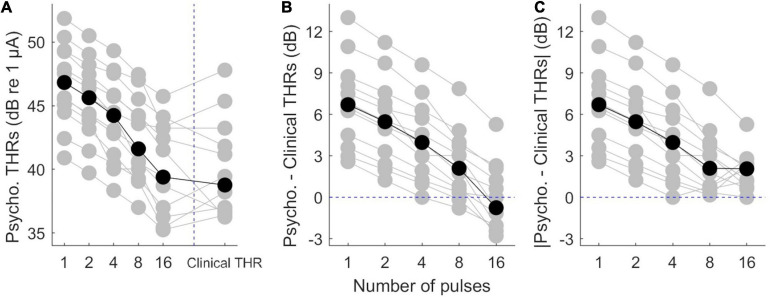
Results of psychophysical THRs. **(A)** Psychophysical THRs as a function of number of pulses. Clinical THRs of all subjects are plotted in the most right side of panel **(A)**. **(B)** The difference between psychophysical THRs and clinical THRs as a function of number of pulses. **(C)** The absolute values of the data in panel **(B)**. The gray circles show results of individual subjects while the black circles show the corresponding median values over all subjects.

Psychophysical thresholds are plotted in Figure 3, where data from individual subjects is plotted in gray while the corresponding median values are plotted in black. Figure 3A shows that while inter-subject variability was high, psychophysical THRs decreased monotonically when the number of pulses increased from 1 to 16. The median THRs dropped from 46.8 dB for a single pulse to 40.4 dB for 16 pulses. Linear regression of psychophysical THRs revealed an average slope of −1.61 dB/doubling the number of pulses. Clinical THRs and their corresponding median values are shown in the right side of Figure 3A. The difference between clinical THRs and the psychophysical THRs, and the corresponding absolute values of the differences are plotted in Figures 3B,C, respectively. This enables us to make a between-subject comparison, and on the other hand, it provides more details on the trend of psychophysical THRs toward clinical THRs.

The median differences between psychophysical THRs and clinical THRs (Figure 3B) decreased monotonically from 6.7 to −0.8 dB when the number of pulses increased from 1 to 16. This is equivalent to a slope of −1.8 dB/doubling the number of pulses. The between-subject range in Figure 3B monotonically decreased from 10.4 to 8.1 dB when the number of pulses increased from 1 to 16. The median of absolute differences between psychophysical THRs and clinical THRs, (Figure 3C) monotonically decreased from 6.70 to 2.10 dB when the number of pulses increased from 1 to 8, which is equivalent to a slope of −1.60 dB/doubling the number of pulses. It further decreased from 2.10 to 2.05 dB when the number of pulses increased from 8 to 16. The between-subject range of the absolute differences monotonically decreased from 10.4 to 5.3 dB when the number of pulses increased from 1 to 16.

### eABR Results

**FIGURE 4:**
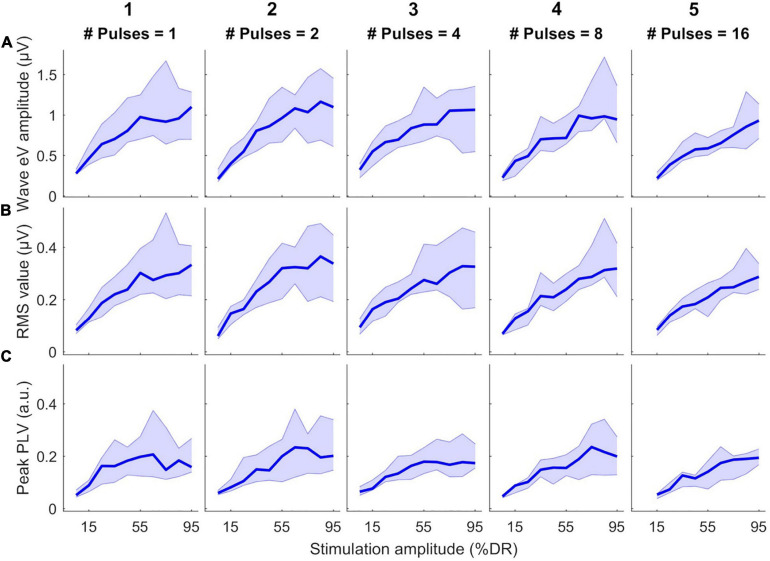
Growth functions of features used to estimate eABR THRs as a function of stimulation amplitude (%DR) in all multiple-pulse (MP) conditions across all subjects. **(A)** Wave eV amplitude, **(B)** eABR RMS value, **(C)** Peak-PLV. Columns show growth functions of the features for specific MP condition. Thick lines show median values and shaded area represents the range between 25th and 75th percentiles.

Figure 4 shows the growth of features as a function of stimulation amplitude (in %DR). Columns 1–5 show growth functions of features in 1-, 2-, 4-, 8-, and 16-pulses conditions, respectively. Rows a-C represent growth functions of wave eV amplitudes, RMS values, and peak PLVs, respectively. The thick lines show the median values of the features over all subjects and the shaded area represents the area between the 25th and 75th percentiles. Wave eV amplitudes were larger than the RMS values and peak PLVs for a given condition. The median RMS values were in most cases larger than their corresponding peak PLVs. Despite having dents, the growth functions of all features showed to be generally monotonic. In some cases, for instance in A3, A4, C3, and C5, the median features saturated at higher stimulation amplitudes. The inter-subject data variability was larger at higher stimulation amplitudes (broader shaded area).

**FIGURE 5:**
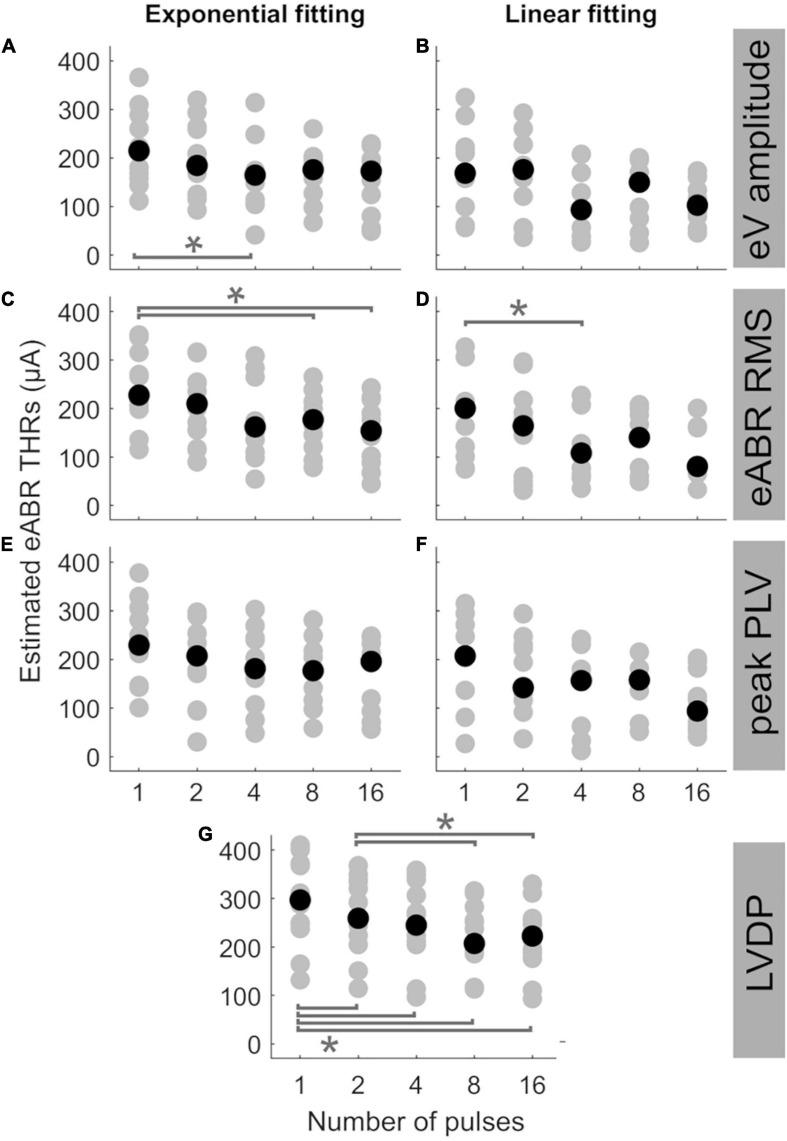
Estimated eABR THRs as a function of number of pulses, resulted from extrapolation of wave eV **(A,B)**, eABR RMS value **(C,D)**, peak PLV **(E,F)**, and LVDP **(G)** growth functions. For each estimation method, an exponential function (left-side panels) and a linear function (right-side panels) were used for fitting. For the LVDP method, no estimation function was used. Data in gray shows results of individual subjects and data in black shows the corresponding median values across subjects. Asterisks show significant differences with α = 0.95.

Figure 5 shows the estimated eABR THRs as a function of number of pulses. The gray circles represent results of individual subjects and the black circles show the corresponding median values across subjects. eABR THRs were estimated by extrapolation of growth functions (panels A–E) or the LVDP (panel F). For each feature, an exponential and a linear function, as described in Eqs 2 and 3, were used to fit the growth function data (left and right panels of Figure 5, respectively). Similar to between-subject difference in psychophysical THRs and DRs, the variability of the estimated eABR THRs was high across subjects. For the eV amplitude feature, eABR THRs in single pulse estimated from exponential fitting functions (FFs) were significantly larger than those in 4-pulses condition, {panel A; [*F*(4, 28) = 5.65, *p* = 0.011]}. For the RMS feature, THRs in single pulse were significantly larger than that in 8-pulses condition {panel C, [*F*(4, 32) = 5.08, *p* = 0.040]}, when estimated from exponential FFs. No significant differences were found for eABR THRs estimated from the peak PLVs. However, for the LVDP feature, more conditions had significantly different estimated eABR THRs. THRs estimated in the single pulse condition were significantly larger than those in the rest of the MP conditions { 2-, 4-, 8-, and 16-pulse conditions; panel G; [*F*(4, 44) = 19.87, *p* = 0.002, *p* = 0.001, *p* = 0.0003, *p* = 0.003], respectively}. Significantly larger THRs were estimated at 2-pulses condition, when compared to 8-, and 16-pulses conditions {panel G, [*F*(4, 44) = 19.87, *p* = 0.001, *p* = 0.0497], respectively}. Note that no extrapolation was used for the LVDP feature.

The median estimated eABR THRs in all panels of Figure 5 decreased when the number of pulses increased from 1- to 4-pulses (no significant differences for individual data). As shown in Table 2, for exponential FFs the median THRs dropped by 50.4, 65.4, and 48.5 μA, respectively for eV amplitude and eABR RMS values, and peak PLV when the number of pulses increased from single pulse to 4-pulses, while for linear fittings the corresponding values dropped by 75.3, 92.2, and 50.9 μA, which suggests larger drops (not significant) for linear FFs when compared to exponential FFs. For the LVDP feature, the median THRs dropped by 51.9 μA when the number of pulses increased from 1 to 4. When the number of pulses increased from 4 to 8, in most conditions the median value increased and then decreased again from 8- to 16-pulses (exceptions were panels E, G). Similar to the comparison between single pulse and 4-pulses conditions, larger (insignificant) drops were observed for linear FFs compared to exponential FFs, when the number of pulses increased from 1 to 16 (details in Table 2).

**TABLE 2:** Median estimated threshold (THR) differences for different features and fitting functions.

Median estimated THR difference between	Feature	Exponential	Linear
1-pulse and 4-pulses (μA)	eV amplitude	−50.1*	−75.3
	RMS value	−65.4	−92.2
	Peak PLV	−48.5	4-50.9
	LVDP	−51.9*	
1-pulse and 16-pulses (μA)	eV amplitude	−42.0	−66.0
	RMS value	−73.3	−119.9
	Peak PLV	−33.6	−113.7
	LVDP	−74.3*	

*Shows statistically significant differences.

Statistical analysis showed that for a given condition in Figure 5, the eABR THRs estimated from exponential FFs were significantly larger than those estimated from linear FFs (worst case *p*<0.04). Due to the inherent nature of the exponential FF compared to the linear FF, the former overestimated the clinical THRs more often than the latter. For wave eV, 85.9% of eABR THR estimates were larger than clinical THRs, when estimated with the exponential FF (panel A), while being 58.3% when estimated with the linear FF (panel B). For RMS feature, the ratio of overestimation for the exponential and linear FFs were 84.5 and 56.3% (panels C, D), respectively, and for peak PLV the ratios were 80.4 and 61.7% (panels E, F), respectively.

**FIGURE 6:**
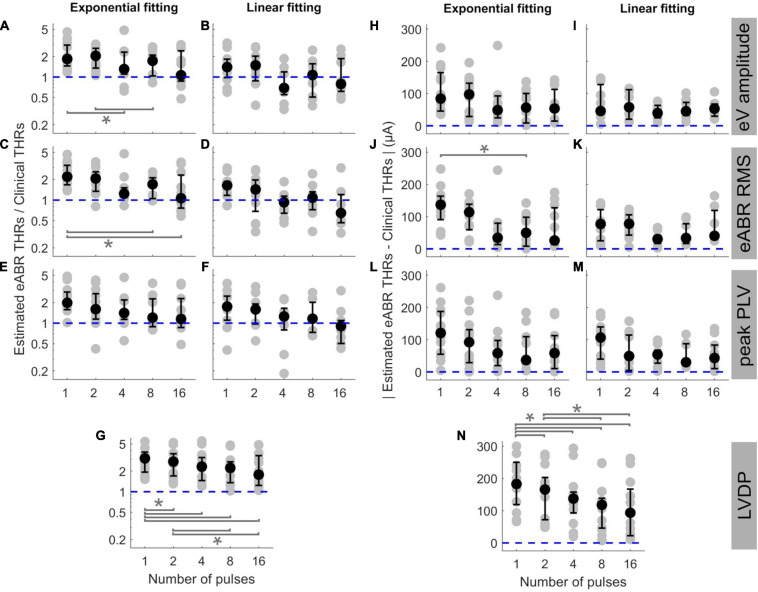
The ratio of estimated eABR THRs to clinical THRs **(A–G)** and absolute difference between them (**H–N**, respectively) as a function of number of pulses. The estimated eABR THRs were presented in Figure 5, which were resulted from extrapolation of growth functions of wave eV **(A,B,H,I)**, eABR RMS value **(C,D,J,K)**, peak PLV **(E,F,L,M)**, and LVDP (**G,N**, without any extrapolation). For each estimation method, an exponential function (left-side panels in each sub-figure) and a linear function (right-side panels in each sub-figure) were used for fitting. For the LVDP method, no estimation function was used. Data in gray shows results of individual subjects and data in black shows the corresponding median values across subjects. Lower and upper error bars show the mean 25th and 75th percentiles of median values from subsamples of growth functions, respectively. Asterisks show significant differences with 0.95 confidence intervals.

In order to examine the predictive power of the estimated eABR THRs presented in Figure 5, we plotted the ratio of estimated eABR THRs to the clinical THRs in Figures 6A–G, as well as the absolute difference between them in Figures 6H–N. The gray circles represent individual THRs and the black circles show the corresponding median values. The lower and upper error bars show the 25th and 75th percentiles, respectively. In Figure 6A (amplitude feature, exponential FF), the ratio of eABR THRs to the clinical THRs in single pulse and 2-pulses conditions were significantly larger than those in 4-pulses [*F*(4, 28) = 4.67, *p* = 0.026] and 8-pulses conditions (*p* = 0.045), respectively. For RMS feature in Figure 6C (exponential FF), the ratio between the two aforementioned THRs at single pulse was significantly larger than those at 8-pulses [*F*(4, 32) = 3.26, *p* = 0.009] and 16-pulses conditions (*p* = 0.033). For RMS features in Figure 6D (linear FF), the ratio at single pulse was significantly larger than those at 8-pulses [*F*(4, 20) = 6.80, *p* = 0.02] and 16-pulses conditions (*p* = 0.03). In panel G (LVDP), where median THR in single pulse condition was significantly larger than that in 2-, 4-, 8, and 16-pulses conditions {[*F*(4, 44) = 23.46, *p* = 0.018, *p* = 0.0003, *p* = 0.0006, and *p* = 0.0002], respectively}, and the median THR in 2-pulses condition was significantly larger than those in 8- and 16-pulses conditions (*p* = 0.001, *p* = 0.012), respectively.

In the absolute difference panels, significant differences were found in panels J (RMS, exponential FF) and n (LVDP). In Figure 6J, the absolute difference between eABR THRs and clinical THRs in single-pulse condition was larger than that in 8-pulses condition [*F*(4, 32) = 3.53, *p* = 0.036]. In panel n (LVDP), significant differences were found between the same pairs as in panel G {larger absolute differences for single-pulse condition compared to 2-, 4-, 8-, 16-pulses conditions: [*F*(4, 44) = 19.87, *p* = 0.002, *p* = 0.001, *p* = 0.0003, and *p* = 0.003], respectively}; larger absolute differences for 2-pulses condition when compared to 8-, and 16-pulses conditions: (*p* = 0.001, *p* = 0.0497). In the ratio panels E-g, the median THRs monotonically decreased as a function of number of pulses, while for absolute difference panels, monotonic decrease of medians was only observed in panel N (LVDP).

The ideal median ratio of 1 did not occur at the same MP condition. In Figures 6A,C,E,F,G, the closest median ratios closest to 1 were found at 16-pulses condition (1.064, 1.065, 1.148, 0.902, and 1.778, respectively), while in Figures 6B,D, the ratios closest to 1 were 1.068 and 0.927. They occurred at 8- and 4-pulses conditions, respectively (details in Table 3). Similarly, in absolute panels of Figure 6, the minimum of median values occurred at different MP conditions. In panels H–K, the minimum median of absolute differences were found in 4-pulses condition (48.6, 39.5, and 30.3 μA, respectively). In panels L and N, the minimum median values were 36.6 and 30.0 μA, respectively, which occurred at 4-pulse condition. In panels J and N, the minimum median values occurred at 16-pulses (25.9 and 93.5 μA, respectively). These results did not consider the between-subject variability and, therefore, might not reflect conditions with the best estimated eABR THRs that applies to majority of the subjects who participated in this study. Table 3 also shows the best conditions for the ratio of eABR THRs to clinical THRs (defined as *A* in Table 3) and the absolute difference between them (defined as *B* in Table 3), with considering the between-subject variability. For relation *A*, the expression *min*⁡|*A*−1| does not account for the between-subject variability, while the expression *min*⁡|*log*⁡*A*| × *mid*50 was introduced to consider it. The variable *mid50* represents the mid 50th percentile (75th percentile–25th percentile). For relation *B*, the expression *min*⁡*B* yields the absolute minimum of the differences between the two THRs, while the expression *min*⁡*B* × *mid*50 would consider the data variability. In Table 3, conditions that minimized the aforementioned expressions are expressed in parenthesis. For a number of cases, conditions with the closest THR estimates remained unchanged, e.g., best conditions in expression *A*, when THRs were estimated with linear FFs. In other cases, however, conditions with the best THR estimates differed when considering the variable *mid50*. For instance, for RMS results and exponential FFs, the minimum of *B* occurred at 16-pulses, while the minimum of *B* × *mid*50 occurred at 4-pulses condition. Data in Figure 6J is in line with this finding, as it suggests that 4-pulses condition would provide smaller median differences and at the same time smaller between-subject variability.

**TABLE 3:** Conditions with the closest electrically-evoked auditory brainstem response thresholds (eABR THRs) to clinical THRs for different features and fitting functions (FFs). For each of relations *A* and *B*, two expressions were defined, one without considering the between-subject variability (***min*⁡|*A*−1|** and ***min*⁡*B***, respectively) and the other with considering it (***min*⁡|*log*⁡*A*| × *mid*50** and ***min*⁡*B* × *mid*50**, respectively). The variable ***mid*50** represents the mid 50th percentile (75th percentile–25th percentile). Conditions (argument) in which the minima of expressions occurred are presented in parenthesis. *nP*, number of pulses.

	$A=\frac{\mathrm{eABR}\mathrm{THRs}}{\mathrm{Clinical}\mathrm{THRs}}$				*B* =|*eABR THRs*−*Clinical THRs*|			
	min⁡|A-1| $\left(\arg\min_{\mathrm{nPulses}}\left|A-1\right|\right)$		min⁡|log⁡A|×mid50 $\left(\arg\min_{\mathrm{nPulses}}\left|\log A\right|\times \mathrm{mid}50\right)$		min⁡B $\left(\arg\min_{\mathrm{nPulses}}B\right)$		min⁡B×mid50 $\left(\arg\min_{\mathrm{nPulses}}B\times \mathrm{mid}50\right)$	
	Exponential	Linear	Exponential	Linear	Exponential	Linear	Exponential	Linear
Amplitude	1.064 (nP = 16)	1.068 (nP = 8)	0.042 (nP = 16)	0.030 (nP = 8)	48.6 (nP = 4)	39.5 (nP = 4)	3315.6 (nP = 4)	1530.6 (nP = 4)
RMS	1.065 (nP = 16)	0.927 (nP = 4)	0.039 (nP = 4)	0.016 (nP = 4)	25.9 (nP = 16)	30.3 (nP = 4)	2295.6 (nP = 4)	774.1 (nP = 4)
PLV	1.148 (nP = 16)	0.902 (nP = 16)	0.086 (nP = 16)	0.026 (nP = 16)	36.6 (nP = 8)	30.0 (nP = 8)	3013.7 (nP = 8)	1968.3 (nP = 8)
LVDP	1.778 (nP = 16)		0.479 (nP = 8)		93.5 (nP = 16)		8921.8 (nP = 4)	

**FIGURE 7:**
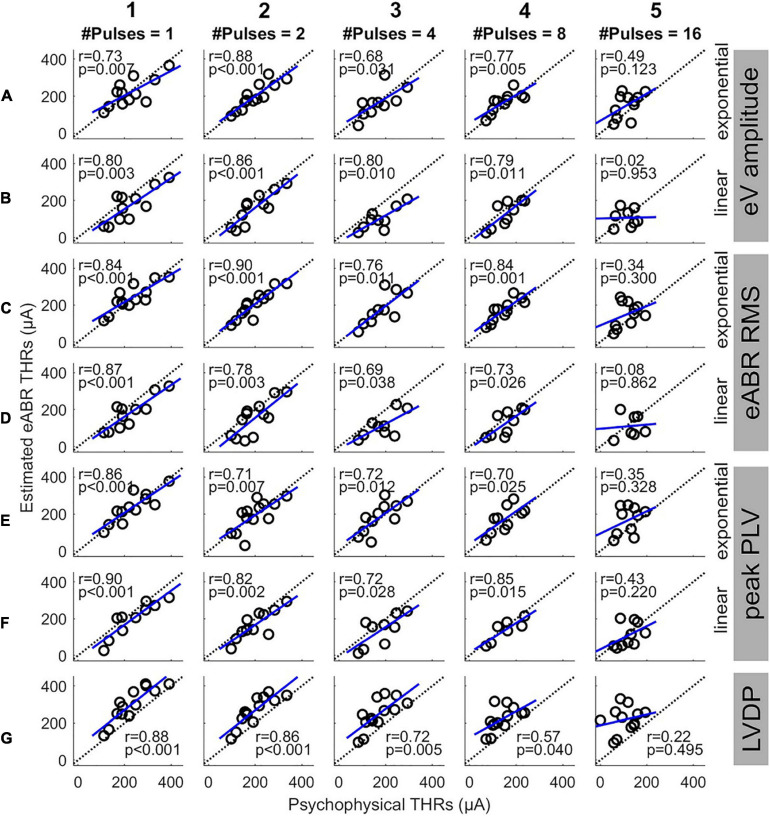
Estimated eABR THRs as a function of psychophysical THRs for multi-pulse conditions. Each column shows results of a specific number of pulses. Panels **(A,B)** show the eABR THRs estimated from AGFs of wave eV with exponential and linear fittings, respectively. Panels **(C,D)** present the eABR THRs estimated from growth functions of eABR RMS values with exponential and linear fittings, respectively. Panels **(E,F)** present the eABR THRs estimated from growth functions of peak PLV with exponential and linear fittings, respectively. Panels **(G)** show the eABR THRs resulted from the LVDP method. Open circles present data from individual subjects. Dotted black lines show the identity lines and the blue lines show linear regressions. In each panel, the correlation coefficient (*r*) and the probability value (*p*) are shown.

Figure 7 shows the eABR THR estimates as a function of psychophysical THRs for all MP conditions and estimation configurations. Individual data are depicted in black open circles. Each row presents eABR THRs resulted for a specific feature and a fitting function and each column shows results for a specific number of pulses. The black dotted lines show lines of equality and the blue lines show linear regressions. In each panel, the PCC (*r*) and the probability value (*p*) are shown. Except for 16-pulse conditions, PCCs were relatively high for the rest of the MP conditions and the corresponding *p*-values showed significance of the correlations. The eABR THRs estimated from linear FFs seem to underestimate the psychophysical THRs when compared to exponential FFs, i.e., data in panels B, D, and F tends to be below the lines of equality. Since no fitting was used for the LVDP feature, the THRs estimated with this feature overestimated the psychophysical THRs, i.e., data in panels G are above the lines of equality. High PCCs in Figure 7 show that it is, in principle, possible to predict behavioral THRs from eABRs. In the 16-pulses conditions, due to the low eABR amplitudes and the lack of enough data points of growth functions, THR estimations were unreliable and could not be performed for all subjects. This also resulted in statistically insignificant PCCs in all 16-pulses conditions. In order to compare the PCCs statistically, they were first transformed to z-scores via Fisher’s *r* to z transformation and then z-test statistics were applied. For a given feature and MP condition, there was no significant differences between the PCCs of the two FFs (for instance, Figures 7C1,D1). For neither exponential nor linear FFs, no significant differences were found between features, e.g., Figure 7A4 compared to C4 or E4). For a given feature and FF, comparison of PCCs of MP conditions showed significant differences only in three pairs (out of 60): 2- and 16-pulses, where RMS feature and exponential FF were used (Figures 7C2,C5), single pulse and 16-pulses, where RMS feature and linear FF were used (Figures 7D1,D5), and 2-, and 16-pulses, where amplitude feature and linear FF were used (Figures 7B2,B5).

**FIGURE 8:**
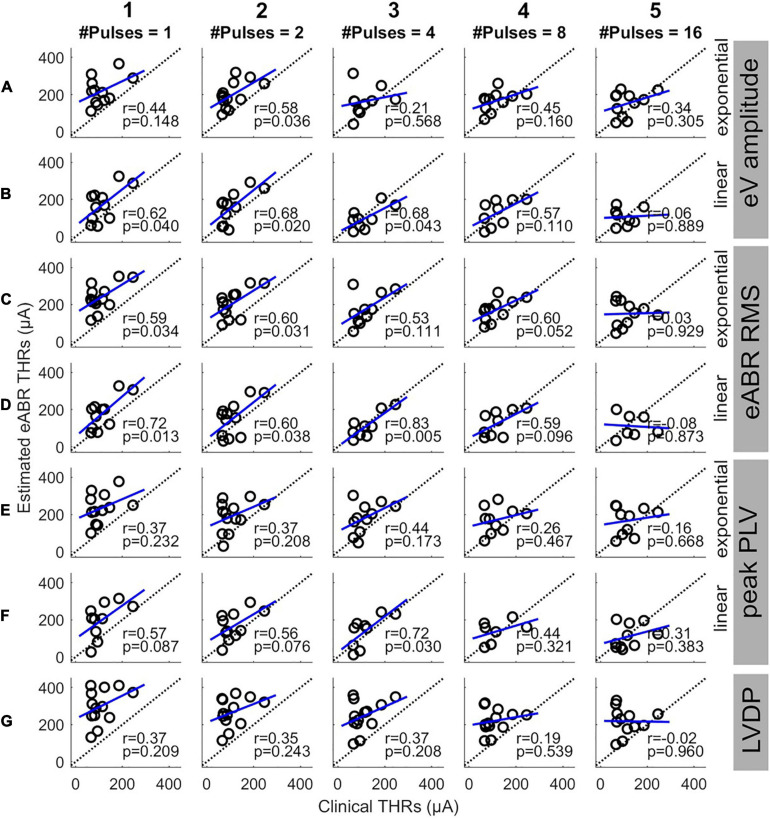
Estimated eABR THRs as a function of clinical THRs for multi-pulse conditions. Each column shows results of a specific number of pulses. Panels **(A,B)** show the eABR THRs estimated from AGFs of wave eV with exponential and linear fittings, respectively. Panels **(C,D)** present the eABR THRs estimated from growth functions of eABR RMS values with exponential and linear fittings, respectively. Panels **(E,F)** present the eABR THRs estimated from growth functions of peak PLV with exponential and linear fittings, respectively. Panels **(G)** show the eABR THRs resulted from the LVDP method. Open circles present data from individual subjects. Dotted black lines show the identity lines and the blue lines show linear regressions. In each panel, the correlation coefficient (*r*) and the probability value (*p*) are shown.

Since this study aimed to estimate clinical THRs, the estimated eABR THRs were plotted as a function of clinical THRs in Figure 8 and correlated. Except for 16-pulse conditions (panels in column 5) and for panel D3, the PCCs in Figure 8 were smaller than their corresponding values in Figure 7. Similar to Figure 7, the PCCs in the 16-pulses conditions were all statistically insignificant and thus were excluded from further analysis. For the linear FFs (panels B, D, and F), the PCCs in the 4-pulses conditions were larger than their corresponding PCCs in the other MP conditions. The largest PCC over all conditions (*r* = 0.83, *p* = 0.005) was resulted from linear fitting of growth functions of the eABR RMS values at the 4-pulses condition (panel D3). Similar to Figure 8, for a given feature and MP condition, comparison of PCCs of the two FFs revealed no significant differences (Figures 8E2,F2). For neither exponential nor linear FFs, no significant differences were found between combinations of the two features, e.g., Figure 8B5 compared to D5 or F5). Finally, for a given feature and FF, comparison of PCCs of MP conditions showed significant differences only between 4- and 16-pulses conditions, where the RMS feature and linear FF were used (Figures 7D3,D5).

## Discussion

The aim of this study was to examine the capability of the eABR THRs in response to MP stimulations to predict clinical THRs. We employed 1-, 2-, 4-, 8-, and 16-pulse burst stimuli with a fast burst rate of 10,000 pps. We found that the behavioral THRs in response to MP stimuli approached clinical THRs when the number of pulses increased from 1 to 16 (Figure 3). Moreover, the between-subject range of the difference between psychophysical THRs and clinical THRs dropped by about 2.3 dB (Figure 3B), and the range of absolute difference by about 4.9 dB (Figure 3C), when the number of pulses increased from 1 to 16. These findings were the motivation to see whether similar findings can be observed in eABR measurements, too. We have extrapolated eABR THRs and evaluated, how well they coincided with clinical THRs and found that MP stimulation protocols indeed provide a better estimate than single pulses, although, inter-subject variability was high. Recording of each stimulation amplitude of MP conditions took about 59 s. If the time spent for subject preparation is excluded from the total measurement time, and if only one MP condition is used to extrapolate THRs, the recording time would be below 10 min (10 stimulation amplitude steps for the AGF × 59 s). This is comparable to the smallest recording time reported by Mao et al. (2018) (12.7 ± 3.1 min), and to the 10-min measurement conditions reported by Mao et al. (2019), but still larger than the 5-min measurement conditions in the same study.

### eABR THR Estimation

Due to the variable trend of the AGFs of electric and acoustic hearing, linear (Mao et al., 2018) and non-linear regressions (Ross et al., 1999; Abbas and Brown, 2015; Visram et al., 2015; Mao et al., 2019) were used for extrapolation of the AGFs. Mao et al. (2018) showed that linear regression can perform equally well when compared to exponential regression. In the study of Mao et al. (2018), the baseline value was defined as the point where the extrapolated FF was zero (x-intercept) while Visram et al. (2015) and Mao et al. (2019) defined the baseline as the point where the extrapolated FF equals to the response to −20 dB sensation level (noise floor). The THRs estimated from the latter were usually larger than those estimated from the former, due to the monotonic increase of the exponential function. In this study, we used growth functions of two time features (wave eV amplitude and RMS value) and one time-frequency feature (peak PLV) to estimate eABR THRs and the zero baseline criterion for THR estimation (Figure 6). As Table 3 suggests, for the two criteria (ratio and absolute difference) the closest eABR THRs to clinical THRs occurred at different MP conditions depending on the two FFs as well as on different expressions used to find the minima. Results in Table 3 showed that without considering the between-subject variability, the MP condition with the closest eABR THRs to clinical THRs could be misleading. As expressions A and B (in Table 3) treat the relation between the estimated eABR THRs and clinical THRs differently, it is not possible to directly compare the corresponding conditions in each expression. The dimensionless expression A cares about the relative difference of the eABR THRs, while expression B considers the absolute linear difference between the two THRs. Presuming two subjects with hugely different hearing DRs, estimation errors of ±50 μA would be treated identically by the expression B, while expression A would treat them differently and more realistically. On the other hand, expression A differentiates underestimations from overestimations, and, thus, it treats them differently. For instance, for a given clinical THR of 50 μA with estimation errors of ±25 μA, expression A would result in ratios of 1.5 (75/50 μA) and 0.5 (25/50 μA), while expression B would treat both errors identically, and assuming an enough large DR, more realistically. Therefore, depending on the THR value, the DR, and required accuracy and sensitivity of estimation, one expression could be used over the other.

In this study, no attempt was made to measure the noise floor in response to stimulus far below the THRs. Therefore, it was not possible to compare the zero baseline method with the noise floor method used for THR estimation. Similar to Visram et al. (2015) and Ross et al. (1999), we defined the THR at a location where the extrapolated FFs were zero. This procedure is responsible for the small underestimation of eABR THRs relative to psychophysical THRs, which can be detected in most conditions of Figure 7. Therefore, when data with more subjects is available, a compensation of this bias could further improve the accuracy of eABR THR extrapolation (Mao et al., 2018).

### eABR THRs vs. Psychophysical THRs and Clinical THRs

In Figures 3B,C, the difference and the absolute difference between psychophysical THRs and clinical THRs monotonically decreased when the number of pulses increased from 1 to 8. From 8- to 16-pulses, the difference further deceased while the absolute difference remained almost the same. Yet, the between-subject range was smaller in the 16-pulse condition when compared to than in the 8-pulse condition. This suggests that psychophysical THRs with 16-pulses could be used as estimations of the clinical THRs with the smallest offset of about 2.0 dB and the smallest between-subject variability of 5.3 dB, when compared to other MP conditions. However, in Figure 6 the absolute difference between estimated eABR THRs and clinical THRs in 16-pulses condition were not always the smallest. This can be explained by the insignificant correlations between psychophysical THRs and estimated eABR THRs (Figure 7, column 5). The fact that eABR THR estimates at 16-pulses failed to show significant correlation with psychophysical THRs could be attributed to two factors: first, the subject-dependent desynchronization of the auditory nerves is largest at 16-pulses condition (especially for the PLV feature), and second, the small number of data points in 16-pulses conditions due to the smaller eABR amplitudes. These reasons severely compromised the precision of THR estimates (right column in Figure 7) and led to large differences to clinical THRs (Figure 6).

Although the PCCs in single pulse conditions of this study (between 0.73 and 0.90) were smaller than those reported in the literature [e.g., *R* = 0.98 in Truy et al. (1998)], we observed that the PCCs were significant and still relatively large for all but 16-pulses conditions (Figure 7). This suggests that up to 8-pulses, the eABR THRs seem to be able to well represent their corresponding psychophysical THRs, and thus, could be able to estimated clinical THRs. As Figure 6 and Table 3 suggest, with considering between-subject variability, eABR THRs in 4- and 8-pulses conditions estimated the clinical THRs better than the other MP conditions. However, as the medium values of the PCCs in Figure 8 shows, eABR THRs in none of MP conditions were able to represent all aspects of the temporal integration elicited by clinical stimuli. The PCCs of the PLV feature in the single pulse condition (0.73 and 0.80, respectively for exponential and linear FFs) were smaller than the PCCs of 0.979 and 0.966 reported in Mao et al. (2019) for Cz-M and Cz-closest montages, respectively. Such a better performance in their study might be due to the fact that they measured responses from more central locations of the auditory pathway, thus resulted in higher correlations between behavioral THRs and CAEP THRs. Since Mao et al. (2019) did not measure clinical THRs (they measured responses to 50-ms electric stimuli), it is not possible to compare the PCCs between clinical THRs and eABR THRs measured from the PLV feature. However, they mentioned two methods to estimate clinical thresholds with longer (500 ms) clinical stimulation: using a correction factor to compensate for the longer stimulation duration, or using longer stimuli, however, these would cause interference with the recorded CAEPs. In this study, we have hypothesized that packing as many pulses as possible within a short stimulation period (longest duration was 1.6 ms) would at least partially consider integration effects and allow us to estimate clinical THRs with higher precision. We plan to extend the multi-pulse stimulation paradigm for CAEP modalities in the future. With the stimulation configuration used in this study, one can pack up to 500 pulses (each of length 100 μs) to construct a 50-ms burst. We assume that measurements with more pulses at more central locations of the auditory pathway could potentially yield even better objective THR estimates.

Fully objective estimation of thresholds of normal and electrical hearing is still challenging from some aspects such as accuracy of the method, measurement equipment, and measurement time. Intra-cochlear measurements, e.g., eCAP, which are provided by the telemetry systems of current implants have their limitations as they can only assess peripheral effects and responses to single pulses. Therefore eCAPs are unable to cover temporal loudness integration, which occurs at higher levels of the auditory pathway. Measurements from mid- to central locations of the auditory pathway usually need additional equipment and longer measurement time to capture more epochs to increase the signal-to-noise ratio. In some measurements such as event-related potential measurements, active listening of participants is required. This makes these methods not applicable to estimate THRs in young babies. However, methods with high precision estimation of THRs are proposed (Visram et al., 2015; Mao et al., 2018, 2019), where they used CAEPs to estimate behavioral THRs. Another issue regarding estimation of behavioral THRs is overestimation, where estimated THRs are larger than behavioral THRs, which reduces the available DR of the CI users.

The eABR seems to be able to estimate behavioral THRs (particularly clinical THRs) at least to some extent. Results of this study show that eABR THRs in response to high-rate multi pulse stimuli could in principle improve objective estimation of clinical THRs. As the stimulation to elicit eABR has to be shorter than some milliseconds to separate stimulation artifacts from eABR responses, loudness integration, which has still longer time constants, cannot be completely covered. Yet, the longer stimulation duration in MP eABRs compared to single pulse measurements (eCAP, single pulse eABRs) at least cover some of the temporal processing aspects and therefore provide a more precise estimation of clinical THRs. Still higher and later potentials might enable even longer lasting stimuli and be able to cover slower effects as loudness integration at more central levels of the auditory system (Abbas and Brown, 2015) with higher precision. On the other hand, higher potentials (CAEPs, eASSR) in young children depend highly on the development of the auditory pathway, which makes their measurement and interpretation harder. However, studies have shown that CAEPs are developed in months-old children (Sharma et al., 2002a,b), with quite stable latencies from birth to age 6, and decreasing P1 and N2 amplitudes and increasing N1 and P2 amplitudes (Wunderlich et al., 2006). It is straight-forward to extend the methods to higher auditory potentials. Our paper provides methods for THR extrapolation of eABRs with extended stimulus durations which should cover higher processing steps as facilitation and, at least partly, temporal loudness integration. These features hold the potential to improve the process of clinical THR estimation with objective measurements.

Accurate clinical THR estimation is important for improvement of CIs’ functionality. Among CI manufacturers, MED-EL and Advanced Bionics set the THRs to 0 clinical units (CU) or 10% of the maximum acceptable levels (Wolfe and Schafer, 2014). The 0 CU would underestimate real THRs while 10% of the MAL could either under- or overestimate the real THRs. In both cases, speech would not be optimally coded within the small DR available for CI users and accurate estimation of the THRs with MP stimulation could improve the performance of the CIs in either case.

In summary, the main contributions of this study are:

• eABRs to bursts of high rate could estimate clinical THRs with smaller errors.
• For the longest pulse trains (1.6 ms, 16 pulses) eABR amplitudes were reduced due to interference, which limited the measurement precision.
• Correlation between eABR THRs and their corresponding psychophysical THRs was generally large (except for 16-pulses) when compared to those between eABR THRs and clinical THRs.
• MP condition at which the smallest difference between eABR THRs and clinical THRs occurred, varied between 4-, 8-, and 16-pulses conditions.

## Data Availability Statement

The raw data supporting the conclusions of this article will be made available by the authors, without undue reservation.

## Ethics Statement

The studies involving human participants were reviewed and approved by the ethics commission of the Klinikum rechts der Isar, Munich (340/19 S). The patients/participants provided their written informed consent to participate in this study. Written informed consent was obtained from the individual(s) for the publication of any potentially identifiable images or data included in this article.

## Author Contributions

AS contributed to the study design, data collection, data analysis, and manuscript drafting. LE contributed to the data collection, data analysis, and manuscript revising. WH contributed to the study design and critical manuscript revising. The final manuscript has been approved by all authors.

## Conflict of Interest

The authors declare that the research was conducted in the absence of any commercial or financial relationships that could be construed as a potential conflict of interest.

## Publisher’s Note

All claims expressed in this article are solely those of the authors and do not necessarily represent those of their affiliated organizations, or those of the publisher, the editors and the reviewers. Any product that may be evaluated in this article, or claim that may be made by its manufacturer, is not guaranteed or endorsed by the publisher.
